# Assessment of the Analytical Capabilities of Inductively Coupled Plasma-Mass Spectrometry

**DOI:** 10.6028/jres.093.107

**Published:** 1988-06-01

**Authors:** H. E. Taylor, J. R. Garbarino

**Affiliations:** U.S. Geological Survey, Denver, CO

A thorough assessment of the analytical capabilities of inductively coupled plasma-mass spectrometry was conducted for selected analytes of importance in water quality applications and hydrologic research. A multielement calibration curve technique was designed to produce accurate and precise results in analysis times of approximately one minute. The suite of elements included Al, As, B, Ba, Be, Cd, Co, Cr, Cu, Hg, Li, Mn, Mo, Ni, Pb, Se, Sr, V, and Zn.

## Experimental

Isotopes for analytical measurements were selected based on freedom from isobaric interferences, whenever possible. Experimental conditions were chosen to minimize both multiply charged and molecular ions.

Samples were run directly, after field filtration (0.45 μm) and preservation with ultrahigh purity nitric acid. To maximize measurement precision, samples were peristaltically pumped to a modified Babington-type nebulizer. Operating conditions of both the plasma and spectrometer are listed in table 1. Optimum ion lens settings varied from element to element as determined by simplex optimization techniques. Compromise settings were selected to facilitate multielement determinations.

Ion currents were measured in the “multielement” mode with a 0.25 second measurement time and 5 replicates. Low resolution conditions were selected with intensity measurements made at 3 points on each peak.

## Results and Discussion

**Figure 1:**
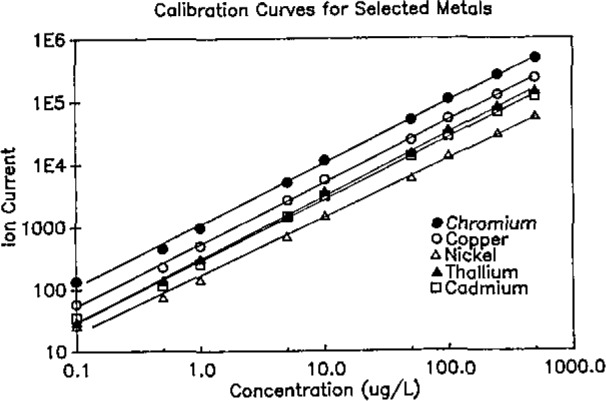
Calibration curves for selected metals.

Representative calibration curves are shown in figure 1 for selected representative elements Cd, Cr, Cu, Ni, and Tl. The curves are linear over at least four orders of magnitude. Similar curves were obtained for each of the other elements. Under these operating conditions, background currents were on the order of 10 counts per second.

Optimally, a coefficient of variation of 5% is obtained at a concentration of approximately 10 μg/L for each element. Below this concentration, the coefficient of variation increases rapidly, at 100 μg/L, it is increased to about 10%.

**Figure 2:**
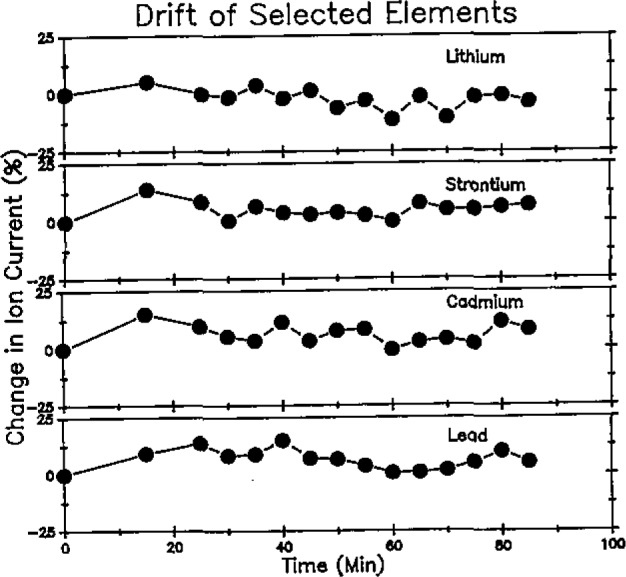
Drift of selected elements.

Drift is shown in figure 2 for representative elements, Cd, Pb, Li, and Sr, which were selected to cover the entire mass range. Drift is shown as a percentage change in ion current from its initial value. This illustration demonstrates that drift across the entire mass range is essentially random in nature and is sufficiently low in magnitude to obviate the need for internal standard compensation.

**Figure 3:**
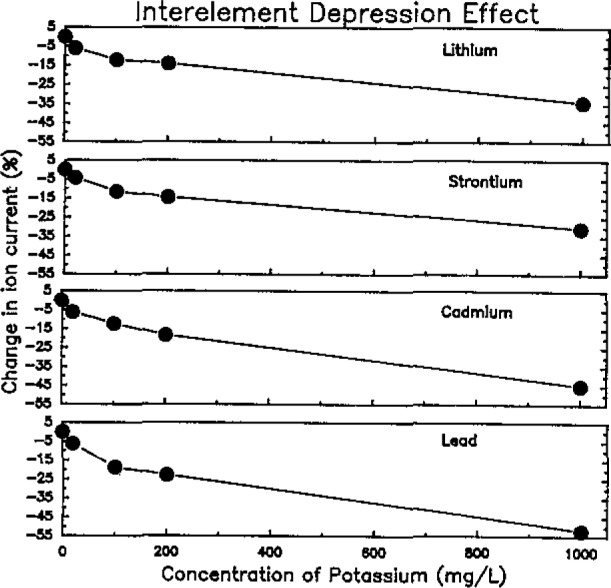
Interelement depression effect.

The effects of sample matrix composition on the accuracy of the determinations showed that matrix elements (such as Na, Ca, Mg, and K) that may be present in natural water samples at concentration levels greater than 50 mg/L resulted in as much as a 10% suppression in ion current for analyte elements. Figure 3 shows the effect of increasing potassium concentration on selected analyte elements, Li, Sr, Cd, and Pb.

Operational detection limits are listed in table 2 for each of the analyte elements. Detection limits were calculated at the 95% confidence level, and represent true detectability for each element.

## Conclusions

Several reference materials were analyzed to determine the degree of bias on the determinations. National Bureau of Standards Standard Reference Materials 1643a and 1643b and an assortment of U.S. Geological Survey Reference Materials were analyzed for each of the elements previously specified. A linear regression of the published values of concentration of these elements versus the experimentally determined concentrations show a linear relationship with a slope of 0.968 and a correlation coefficient of >0.99.

The data and examples presented show that the technique described is a suitable approach for analyzing natural environmental water for trace elements.

## Figures and Tables

**Table 1:** Operating parameters

RF Power	1.2 kW
Plasma Ar flow	13 L/min
Aux. Ar flow	1.4 L/min
Aerosol Ar flow	0.5 L/min
Sample delivery	1.8 mL/min
Ion lenses	
Barrel	4.3 V.D.C.
Stop	−5.2 V.D.C
Einzels	−13 V.D.C.
Plate	−14 V.D.C.

**Table 2:** Detection limits (μg/L)

Element	Det. limit	Element	Det. limit
Ag	0.8	La	0.1
Al	1	Li	0.4
As	0.5	Mn	0.2
B	0.8	Mo	0.4
Ba	0.5	Ni	0.3
Be	0.4	Pb	0.4
Cd	0.6	Se	0.2
Co	0.1	Sr	0.2
Cr	0.6	Sn	2
Cu	0.3	Tl	0.2
Hg	7	V	0.5
		Zn	0.7

